# Relative quantification of salt soluble allergens in wheat by targeted mass spectrometry

**DOI:** 10.1186/2045-7022-5-S3-P132

**Published:** 2015-03-30

**Authors:** Marija Pavlovic, Roberta Lupi, Sandra Denery-Papini, Olivier Tranquet, Helene Rogniaux, Colette Larré

**Affiliations:** 1 Institut National de la Recherche Agronomique, UR1268 BIA, Nantes, France

## 

Wheat is an important part of the daily diet of millions of people. However, this staple food is also responsible for IgE mediated food allergies. Until now, a life-long wheat-free diet has been the only causal therapy for people suffering from these pathologies. In this context, it is of paramount interest to develop analytical methods able to detect and quantify the wheat allergens in complex food matrices, with a good sensitivity and reliability. Mass spectrometry offers an interesting alternative to immune-detection, with the main advantage over ELISA that it enables to monitor several allergens within a single analysis, with the high specificity and sensitivity available on modern instruments.

Fifteen allergens of the wheat soluble protein extracts were targeted in this study. After in silico hydrolysis, 30 proteotypic peptides were retained to be monitored in the Orbitrap mass spectrometer using a pseudotargeted MS/MS method. Ion transitions were predicted for each of the proteotypic peptides by Skyline software and validated experimentally on our samples. Run-to-run variations in signal intensities were corrected by normalizing the signal against an external peptide.

This method permitted the monitoring of fourteen wheat allergens of the albumin/globulin fraction in a single experiment. The short sequence of some of these allergens added to their high level of similarity reduced the choice for a proteotypic peptide and its transition ions; some of them were quantified only with one precursor. Relative quantification was performed between six varieties originating from three species (T. monococcum, T. durum and T. aestivum).

The pseudo targeted MS/MS method developed in this study for analysis of whole grain salt soluble extracts enabled the relative comparison of the content of salt soluble allergens in wheat cultivars. Further developments are being implemented in order to reach quantitative analysis.

